# Design of a Clinical Decision Support System for Fracture Prediction Using Imbalanced Dataset

**DOI:** 10.1155/2018/9621640

**Published:** 2018-03-22

**Authors:** Yung-Fu Chen, Chih-Sheng Lin, Kuo-An Wang, La Ode Abdul Rahman, Dah-Jye Lee, Wei-Sheng Chung, Hsuan-Hung Lin

**Affiliations:** ^1^Department of Radiology, BenQ Medical Center, The Affiliated BenQ Hospital of Nanjing Medical University, Nanjing, Jiangsu Province 210017, China; ^2^Department of Healthcare Administration and Department of Dental Technology and Materials Science, Central Taiwan University of Science and Technology, Taichung 40601, Taiwan; ^3^Department of Health Services Administration, China Medical University, Taichung 40402, Taiwan; ^4^Department of Electrical and Computer Engineering, Brigham Young University, Provo, UT 84602, USA; ^5^Department of Industrial Education and Technology, National Changhua University of Education, Changhua County 50007, Taiwan; ^6^Department of Management Information Systems, Central Taiwan University of Science and Technology, Taichung 40601, Taiwan; ^7^Department of Internal Medicine, Taichung Hospital, Ministry of Health and Welfare, Taichung 40343, Taiwan

## Abstract

More than 1 billion people suffer from chronic respiratory diseases worldwide, accounting for more than 4 million deaths annually. Inhaled corticosteroid is a popular medication for treating chronic respiratory diseases. Its side effects include decreased bone mineral density and osteoporosis. The aims of this study are to investigate the association of inhaled corticosteroids and fracture and to design a clinical support system for fracture prediction. The data of patients aged 20 years and older, who had visited healthcare centers and been prescribed with inhaled corticosteroids within 2002–2010, were retrieved from the National Health Insurance Research Database (NHIRD). After excluding patients diagnosed with hip fracture or vertebrate fractures before using inhaled corticosteroid, a total of 11645 patients receiving inhaled corticosteroid therapy were included for this study. Among them, 1134 (9.7%) were diagnosed with hip fracture or vertebrate fracture. The statistical results showed that demographic information, chronic respiratory diseases and comorbidities, and corticosteroid-related variables (cumulative dose, mean exposed daily dose, follow-up duration, and exposed duration) were significantly different between fracture and nonfracture patients. The clinical decision support systems (CDSSs) were designed with integrated genetic algorithm (GA) and support vector machine (SVM) by training and validating the models with balanced training sets obtained by random and cluster-based undersampling methods and testing with the imbalanced NHIRD dataset. Two different objective functions were adopted for obtaining optimal models with best predictive performance. The predictive performance of the CDSSs exhibits a sensitivity of 69.84–77.00% and an AUC of 0.7495–0.7590. It was concluded that long-term use of inhaled corticosteroids may induce osteoporosis and exhibit higher incidence of hip or vertebrate fractures. The accumulated dose of ICS and OCS therapies should be continuously monitored, especially for patients with older age and women after menopause, to prevent from exceeding the maximum dosage.

## 1. Introduction

Chronic respiratory diseases, including chronic obstructive pulmonary disease (COPD), asthma, bronchiectasis, allergic rhinitis and sinusitis, obstructive sleep apnoea syndrome, pulmonary hypertension, and other occupational lung diseases, are caused by disorder of the airways and other structures of the lung [1]. More than 1 billion people suffer from chronic lung diseases, accounting for more than 4 million deaths annually [2]. Among them, more than 200 million people were afflicted by COPD causing 3 million deaths globally; more than 235 million people were affected by asthma resulting in 0.18 million deaths [3, 4] with over 80% of deaths found in countries with low- and lower-middle income [5]; and the incidence rate of bronchiectasis ranged from 4.2 per 100000 persons aged 18–34 years to 272 per 10000 persons aged 75 years and above in the US [6] with an increasing rate of 8.7%/year from 2001 to 2007 [7].

COPD is an inflammatory disease of the lung characterized by progressive airflow obstruction, systematic chronic inflammation, and recurrent acute exacerbation [8, 9]. WHO predicted that COPD will become the 3rd leading cause of death worldwide in 2030 [10]. Bronchiectasis is characterized by abnormal bronchial dilation and bronchial wall thickness, chronic infection and inflammation, recurrent cough and sputum production, and bacterial colonization and airflow obstruction, resulting in a decline in respiratory function [11]. Compared to other chronic diseases, asthma has a relatively lower fatality rate. Asthma is characterized by chronic airway inflammation with the history of respiratory symptoms like wheeze, shortness of breath, chest tightness, and cough accompanied with expiratory airflow limitation [12]. Oral corticosteroids (OCS) and inhaled corticosteroids (ICS) are usually prescribed by physicians to improve symptom, lung function, and quality of life, as well as to reduce repeated exacerbations for patients with asthma, COPD, and bronchiectasis. However, it was reported that corticosteroid use may increase the risk of fracture [13–15].

Clinical decision support systems (CDSSs) provide useful information and expert knowledge to assist healthcare providers to improve diagnosis and treatment outcomes, disease managements, and healthcare quality for patients in both home and clinical settings and have been shown to be capable of improving healthcare outcomes in medical practice [16]. CDSSs have been widely applied in disease diagnosis [17, 18], disease treatment and management [19–21], medical alerting or event reminding [22–24], and drug dosing or medication prescribing [25, 26].

### 1.1. Problems Encountered in CDSS Design with Imbalanced Datasets

Taiwanese National Health Insurance program is a single-payer, compulsory insurance system that was established in 1995 by the Bureau of National Health Insurance (NHI), Ministry of Health and Welfare. The insurance program provides healthcare to 99% of the 23.74 million citizens of Taiwan and maintains contracts with 97% of the nation's healthcare institutions. The National Health Research Institute is authorized to establish the National Health Insurance Research Database (NHIRD), as well as to manage registration and claim data for the 23 million insured citizens. Most studies used a subset of the NHIRD that consisted of 1 million randomly sampled beneficiaries enrolled in the NHI program. According to a PubMed website search conducted in July 2016, around 4000 studies investigated using NHIRD have been published [27]. The high accuracy and validity of ICD-9-CM diagnoses in the NHIRD have been described in previous studies [28]. Recently, we have conducted several studies to discover the association between an individual disease with the risk of acquiring another diseases based on the data retrieved from the NHIRD, for example, the investigation of inhaled corticosteroids with pulmonary tuberculosis [29], sleep disorders with erectile dysfunction [30], gout with erectile dysfunction in men [31], and bronchiectasis with ischemic stroke [32]. Because most of the datasets retrieved from the NHIRD for the above-mentioned studies are imbalanced with ratios of positive samples (minority) to negative samples (majority) ranging from 1 : 4 to 1 : 5, the decision hyperplane will bias toward the majority class when adopting the accuracy-driven algorithm to design the CDSS.

Minority cases refer to rare patterns or abnormal behaviors that are difficult to detect but are often important. In real application domains, such as oil spill detection in satellite radar images [33], text classification [34], financial fault detection [35, 36], customer identification [37], medical diagnosis [38, 39], and others, classification of imbalanced datasets incurs critical problems. One problem encountered in classifying unbalance datasets is that samples of one class (majority) outnumber the samples in another class (minority) that is of often more interest or importance, making the algorithms driven by accuracy bias toward the majority class [40]. Generally, although the classification accuracies are satisfactory in the classifiers designed based on the accuracy-driven (treating accuracy as the fitness function) algorithms, their sensitivities are quite low. Hence, alternative fitness functions, such as area under ROC curve (AUC) [41] or weighted sum of accuracy, sensitivity, and specificity, are proposed to solve these problems.

### 1.2. State-of-the-Art Methods for CDSS Design

The classifiers built based on logistic regression, decision tree, standard neural network, and support vector machine are generally suitable for balanced data only. When dealing with imbalanced data, these classifiers often bias toward the majority cases while distorting the minority cases. To improve the predictive performance, the methods for modeling imbalanced datasets include data preprocessing, cost-sensitive learning, and kernel-based methods [42].

Resampling data in the sample space and selecting features in the feature space are commonly used preprocessing strategies for dealing with imbalanced datasets. There are 3 categories of resampling methods, including undersampling, oversampling, and hybrid sampling methods, applied for rebalancing the imbalanced datasets. Oversampling methods are used to deal with the minority samples by generating new minority samples, while undersampling strategies are applied to randomly discard the majority samples to balance the datasets. The hybrid sampling is a combination of both methods.

Undersampling includes random undersampling and informed undersampling [42]. The major drawback of undersampling is that important information may be lost due to the removal of some data points. There is no specific mechanism under random undersampling, which only functions randomly. Other undersampling methods like one-sided selection [43], BalancedCascade, and EasyEnsemble [44] are called informed undersampling. One-sided selection obtains the balanced training set from an imbalanced one by keeping the minority samples untouched while selectively removing the borderline majority samples by applying Tomek link concept [43]. It eliminates boundary samples and deals with only a subset of majority samples. Devi et al. [45] proposed a modified Tomek link-based undersampling scheme to eliminate, besides boundary samples, outlier and redundant samples to improve the one-sided selection method. In EasyEnsemble and BalancedCascade, several subsets of training data are sampled from the majority samples and each subset is combined with the minority samples for training a model. The generated models are then combined for making the final decision [44].

The simplest oversampling method is random oversampling, in which the minority samples are randomly duplicated. A critical problem of random oversampling is overfitting. Another major approach in oversampling is synthetic minority oversampling technique (SMOTE), which generates synthetic samples on the line segments connecting each minority sample to its k-nearest neighbors in minority class [46]. A major problem of SMOTE is that it blindly generates synthetic samples without considering the majority data points located close to the minority samples, resulting in overlapping between classes. Later, several methods, such as Borderline-SMOTE [47], Safe-Level-SMOTE [48], and Cluster-SMOTE [49], extending the conventional SMOTE were proposed to improve the performance.

Cost-sensitive learning is useful for handling imbalanced healthcare data since classifying a minority (positive) sample to the majority (negative) class often costs more than classifying a majority sample to the minority class. There are 3 major categories generally found in cost-sensitive learning: approaches that assign different weights to samples, ensemble schemes that integrate with cost-sensitive methods, and methods that incorporate the misclassified costs directly into the classifiers [42]. The first approach is motivated by the AdaBoost scheme which trains an initial model based on the original imbalanced dataset and identifies misclassified samples. More weight will be assigned to the misclassified samples in the following iterations until the classifier significantly improves. In the second scheme, boosting approach is generally used for improving the imbalanced data problem and multiple base learners are trained to solve the classification problem. Many types of ensemble learning methods like bagging, boosting, and stacking have been proposed to combine base learners according to different strategies. In the third method, by considering costs of misclassification differ among different classes, the classifier is designed by minimizing the total misclassification cost. In the current study, the strategy for designing the CDSSs is to weight more on sensitivity in the objective function or to maximize the AUC for achieving higher sensitivity in detecting more patients (minority cases) to increase their well-being.

Support vector machine (SVM) is mostly integrated in kernel-based methods for imbalanced data learning. In Farquad and Bose [40], oversampling of the minority class was achieved by training an SVM model with lower accuracy but higher sensitivity by increasing the value of SVM model parameter *C* to convert the misclassified majority samples into minority samples. The reconstructed more balanced training dataset was then applied for training the classification models with better predictive performance using different artificial techniques like multilayer perceptron (MLP), logistic regression (LR), and random forest (RF). In contrast, Jian et al. [50] applied the biased SVM to identify the support vectors and nonsupport vectors of the imbalanced training samples and then used SMOTE and random undersampling methods to resample the support vectors in the minority class and nonsupport vectors in the majority class, respectively. Recently, Piri et al. [42] proposed a new SMOTE algorithm by oversampling the informative minority samples near the SVM decision boundary. Additionally, they focused on misclassified informative minority samples by oversampling them with a higher degree than the correctly classified minority samples. The algorithm generates much less synthetic samples and is more efficient than SMOTE [46], Borderline-SMOTE [47], Safe-Level-SMOTE [48], and Cluster-SMOTE [49].

According to the aforementioned description, the datasets retrieved from the NHIRD for public health studies are mostly imbalanced with minority to majority sample ratios ranging from 1 : 4 to 1 : 5, and the decision hyperplane tends to bias toward the majority class when adopting the accuracy-driven algorithm to design the CDSS. The dataset adopted in this study is even more imbalanced with the minority to majority ratio approximates to 1 : 9. The objectives include the following: (1) investigate the association between corticosteroid use and fracture using NHIRD and (2) design the CDSSs to predict fracture occurrence for patients with chronic respiratory diseases prescribed with corticosteroids by dealing with the imbalanced dataset. The preliminary results were reported in [51].

## 2. Materials and Methods

### 2.1. Data Sources

Data of the patients who were 20 years old or older visiting healthcare centers (outpatients or inpatients) and had been prescribed with inhale corticosteroids because of diagnosed asthma (ICD-9-CM 493), chronic obstructive pulmonary disease (COPD) (ICD-9-CM 491, 492, and 496), or bronchiectasis (ICD-9-CM 494) within 2002–2010 were retrieved from the NHIRD for this investigation. Patients diagnosed with hip or vertebrate fractures before using inhaled corticosteroid were excluded. The outcome measure was hip fracture (ICD-9-CM 820) or vertebral fracture (ICD-9-CM 805 and 806). The data of 11645 patients, including 1134 patients with the fracture (614 men and 520 women) and 10511 patients without fracture (6211 men and 4300 women), were retrieved from the NHIRD within 2001–2013 and were used for statistical analysis and CDSS design. The patients were divided into 4 groups according to age: 20–40, 41–50, 51–64, and ≥65 years. The respiratory diseases included were asthma (ICD-9-CM codes 493), COPD (ICD-9-CM codes 491, 492, and 496), and bronchiectasis (ICD-9-CM codes 494). Other comorbidities included diabetes (ICD-9-CM codes 250), cancer (ICD-9-CM codes 140–208), liver cirrhosis (ICD-9-CM codes 571.2 and 571.5), end-stage renal disease (ICD-9-CM codes 585), and osteoporosis (ICD-9-CM codes 722.0).

SPSS 22.0 software (IBM.SPSS) was adopted for all statistical analyses. Difference in the proportional distribution of the demographic characteristics and comorbidities of the patients with fracture and those without fracture were compared and tested using the Chi-square test. The overall, age-specific, and comorbidity-specific incidences of fractures in both fracture and nonfracture groups were also compared. The cumulative dose, mean follow-up daily dose, and mean exposed daily dose of prescribed corticosteroid were also analyzed using unpaired Student's *t*-test. The statistical significance was defined as *p* < 0.05.

### 2.2. Design of Clinical Decision Support Systems

**Figure 1:**
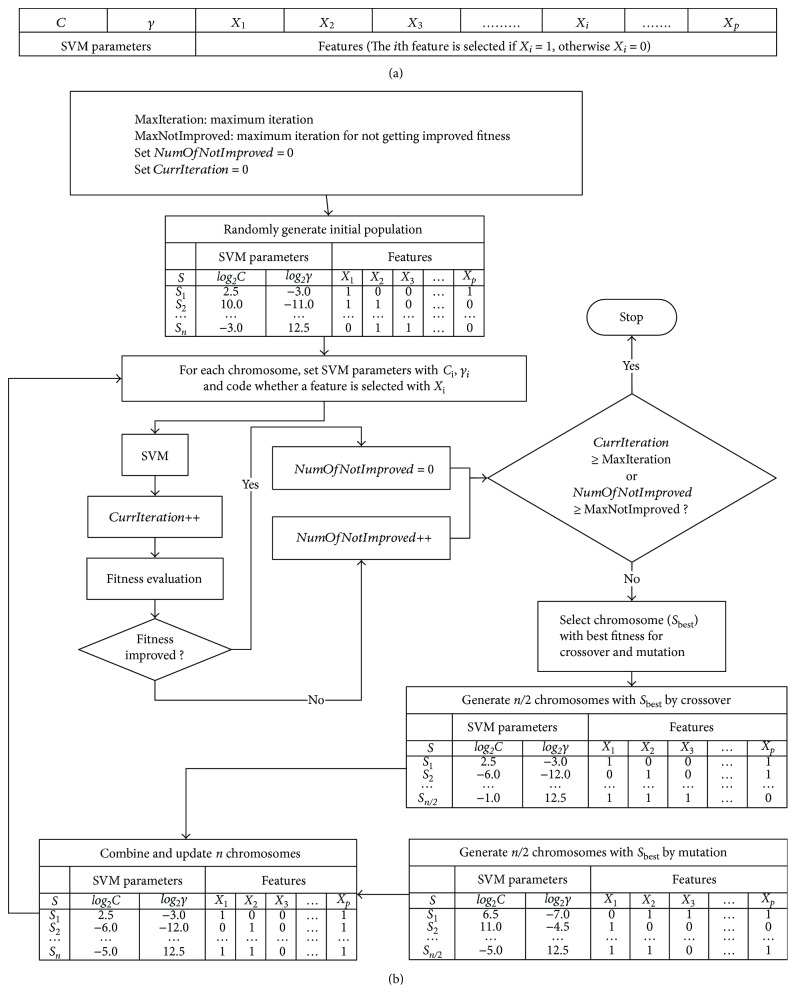
The wrapper method combining genetic algorithm and SVM: (a) a chromosome example and (b) the flowchart of the adopted wrapper method with genetic algorithm used for selecting features and adjusting SVM parameters as well as SVM for classifying different classes and calculating fitness values based on the objective functions.

As shown in Figure 1, a wrapper method integrating genetic algorithm (GA) and support vector machine (SVM) was used for designing the CDSSs with the former adopted for selecting salient features and adjusting the SVM parameters (cost value and kernel parameter) whereas the latter for classifying different classes and calculating fitness values based on the objective functions [19]. For each iteration, the *n* chromosomes were updated by combining *n*/2 new chromosomes generated from crossover with the other *n*/2 chromosomes obtained from mutation. The parameters of the genetic algorithm were set as follows: number of initial chromosome population (*n*) = 10, maximum number of iterations with unimproved fitness value (MaxNotImproved) = 500, and maximum number of total iterations (MaxIteration) = 100000. When the current iteration (CurrentIteration) ≥ MaxIteration or the number of iterations with unimproved fitness (NumOfNotImproved) ≥ MaxNotImproved, the program terminated.

#### 2.2.1. Data Preprocessing and Preparation

The data retrieved from the NHIRD were divided into training and testing datasets, each contains 50% of the samples in majority class and minority class, respectively, that is, 567 samples with fracture and 5255 samples without fracture. Random undersampling, cluster-based undersampling [52], and one-sided selection [43] methods were used to prepare the training datasets before designing the CDSSs. After random undersampling, the balanced training set consisted of 2*m* samples by including all the *m* minority samples and randomly selecting *m*-nonduplicated samples from the *M* (*M* > *m*) samples in the majority class. In contrast, in the cluster-based undersampling, the samples in the majority class were clustered into *m* clusters that each consisted of ⌊*M*/*m*⌋ or ⌊*M*/*m*⌋ + 1 samples. And then, the kNN algorithm was applied to select the sample which was nearest to the center of gravity in each cluster, resulting in a balanced training set containing 2*m* samples for cross-validation. After the CDSSs had been trained, the imbalanced testing dataset was used for testing the predictive performance of the models.

#### 2.2.2. Model Training and Testing

Independent training and testing (ITT) was adopted for training and testing effectiveness of the CDSS [53] that 50% of the data were used for training and validating and the rest 50% for testing its predictive performance by calculating the accuracy, sensitivity, specificity, G-mean, and AUC. In the training phase, tenfold cross-validation was adopted for training and validating the models for obtaining a model with the best performance. For cross-validation, all the sample data in the training set were randomly divided into 10 clusters (folds), in which any combined nine folds were used for training while the remaining one for validating the CDSSs. The procedure was repeated for 10 times.

In the testing phase, the imbalanced testing dataset was applied for testing the designed CDSSs obtained in the testing phase. When designing the CDSSs, selection of the objective function is crucial in obtaining optimal CDSS models. In this study, the cost-sensitive objective functions, including AUC and combined accuracy, sensitivity, and specificity, were used to obtain the optimal CDSSs with imbalanced datasets. Equation (1) shows the objective function which combines the accuracy, sensitivity, and specificity. Equation (2) shows the AUC treated as the objective function.

(1) $$\begin{array}{c} OB1=AC-\left|SE-SP\right|, \end{array}$$

(2) $$\begin{array}{c} OB2=AUC. \end{array}$$

In (1), we intended to maximize the value of the objective function by increasing the accuracy (AC) and decreasing the difference between the sensitivity (SE) and specificity (SP) at the same time to avoid the decision hyperplane to bias toward the majority class, whereas in (2), only AUC is maximized.

In order to verify the feasibility of the proposed method, a pilot study which used the CoIL challenge dataset [40] and WDBC dataset [54] was conducted and compared with the results obtained in previous studies [40] and one-sided selection [43], respectively.  Table 1 shows the predictive performance of the CDSSs trained by using training datasets obtained by random undersampling. As shown in the table, the predictive performances of the 10 training subsets are very similar with an average predictive accuracy, sensitivity, specificity, G-mean, and AUC of 66.17%, 62.85%, 66.38%, 0.6439, and 0.7069, respectively.

Table 2 compares the predictive performance among CDSS models designed using different methods based on the CoIL challenge dataset. As shown in the table, unlike biases exhibited in models designed with accuracy-driven algorithms, the method proposed in this study presents higher AUC with more unbiased sensitivity and specificity than the methods proposed in [40], that is, SVM+ 100% oversampling, MLP + SMOTE, hybrid SVM-MLP + 100% oversampling, LR + SMOTE, and hybrid SVM-LR + 100% oversampling, as well as decision tree (J48) + cluster-based kNN undersampling and one-sided selection. Notice that the one-sided selection methods achieve 0% sensitivity and 100% specificity in the testing phase for its inability of obtaining a balanced training set after undersampling (5474 majority samples and 348 minority samples before and 5236 majority samples and 348 minority samples after one-sided selection).

Table 3 compares the predictive performance among the CDSS models designed based on our proposed methods and the one-sided selection methods using the WDBC dataset. As noted in the table, the predictive performance of the models designed with one-sided selection methods demonstrates similar performance with models built using our proposed methods. Compared with the one-sided selection methods, the results indicate that our proposed methods are more robust with the predictive performance less influenced by different datasets adopted for designing CDSSs.

## 3. Results and Discussions

### 3.1. Statistical Analysis

Comparisons of demographic characteristics, comorbid respiratory diseases, and other comorbidities between the patients with and without fracture are shown in Table 4. As shown in the table, age and gender distributions of the patients in fracture cohort and comparison cohort were significantly different (*p* < 0.01). The patients with fracture were older than those without fracture (*p* < 0.001). The mean ages of the patients with fracture were 70.5 ± 12.5 years, and those without fracture were 58.5 ± 18.1 years. The prevalence of respiratory diseases and other comorbidities, including COPD (64.7% versus 51.6%), DM (14.9% versus 12.4%), end-stage renal disease (2.8% versus 1.4%), and osteoporosis (7.1% versus 3.6%), was significantly higher in the fracture cohort than in the comparison cohort (*p* < 0.05), while the prevalence of asthma was significantly lower in the fracture cohort than in the comparison cohort (*p* < 0.001). On the other hand, bronchiectasis (5.8% versus 4.7%), liver cirrhosis (1.1% versus 0.8%), and cancer (4.7% versus 4.4%) were not significantly different between the 2 groups (Table 4).

Table 5 exhibits the association between prescribed oral or intravenous corticosteroid and fracture. The cumulative dose (2355.86 ± 4049.43 versus 1345.77 ± 2831.30) and mean exposed daily dose (94.04 ± 314.98 versus 67.65 ± 235.62) of patients with fracture were significantly higher than the nonfracture cohort (*p* < 0.01), while the mean follow-up daily dose (59.56 ± 285.58 versus 59.74 ± 218.39) did not reach significance (*p* > 0.05). Regarding the follow-up (999.65 ± 997.09 versus 706.77 ± 907.35 days) and exposed (154.04 ± 320.91 versus 90.11 ± 194.80 days) durations, the patients with fracture were significantly longer (*p* < 0.001) than those without fracture.

Table 6 shows the association between prescribed inhaled corticosteroid and fracture. As indicated in the table, the cumulative dose (230.14 ± 442.76 versus 171.97 ± 318.13) of patients with fracture was significantly higher than the patients without fracture (*p* < 0.001), while the mean follow-up daily dose (0.44 ± 2.31 versus 1.00 ± 3.91) was significantly lower (*p* < 0.001). On the other hand, the mean exposed daily dose (0.51 ± 0.81 versus 0.55 ± 1.35) was not significantly different between patients with and without fracture (*p* > 0.05). With regard to the follow-up (1777.17 ± 1039.82 versus 1370.91 ± 1097.76 days) and exposed (453.79 ± 584.65 versus 342.35 ± 503.32 days) durations of inhaled corticosteroid, the patients with fracture were significantly longer (*p* < 0.001) than those without fracture.

### 3.2. Clinical Decision Support System

Table 7 compares the predictive performance in both training and testing phases of the CDSSs designed using balanced training sets obtained from different undersampling methods.  Table 8 shows the optimal SVM parameters and selected features for CDSS design.

### 3.3. Discussions

Although allergic rhinitis and sinusitis are also a type of chronic respiratory diseases, they are not included for analysis in the current study for they are usually treated with intranasal corticosteroid [55] having lower concentration and smaller effect than oral or inhaled corticosteroids [56]. Neither pulmonary hypertension nor occupational lung diseases are not treated with corticosteroid. It was observed that intranasal corticosteroid suppresses children's growth in short-term study [57], but not affecting adult height for children in long-term treatment [58]. Among the elderly people, administration of low-dose inhaled corticosteroids exhibited small but significant excess risk of cataracts but not observed in use of nasal corticosteroids [56]. Patients with asthma who received the ICS were reported to have higher risk of contracting pneumonia or lower respiratory infection [59] and have effect on adult height in children [60]. Similar to asthma, patients with COPD receiving ICS therapy also exhibited increased risk of pneumonia [61] and TB [62].

As shown in Table 4, the majority of patients with fractures in this study were men (54.1%) with mean age of 70.5 ± 12.5, which is similar to the results obtained from some previous studies, for example, 94% (mean age: 62.7 ± 12.4) reported in [63] and 60% (mean age: 61.2 ± 9.0) in [64], while contradicting to another study conducted by Pujades-Rodríguez et al. [15] with only 40% (mean age = 69.3 ± 10.03) were male in patients with COPD.

#### 3.3.1. Effects of OCS and ICS on Fracture

OCS was reported to be associated with an increased dose-response risk of fracture or osteoporosis in patients with chronic respiratory disease [65] and patients with asthma [66]. Long-term use of OCS may also induce other adverse effects; for example, it was reported that morbidity rates for patients with severe asthma comorbid with other diseases, such as type-2 diabetes, dyspeptic disorder, and cataract, prescribed with higher OCS dose were significantly higher than those with mild/moderate asthma. Glucocorticoid highly decreases bone formation by inhibiting cell differentiation, and increasing apoptosis might be the mechanism causing such an effect [67]. Consistent to previous investigations, our study showed that the cumulative doses of OCS and ICS were significantly higher for patients with fracture than those without fracture, indicating the dose-response effects (Tables 5 and 6). Whether ICS use is associated with osteoporosis or fracture is still controversial. Some previous studies supported that ICS use increased the risk of fracture in patients with COPD [13–15] and patients with asthma [68], while others were against the above conclusion in COPD [65, 69] and asthma [70]. Differences in study design, duration of ICS use and cumulative dose, and frequency of systemic OCS prescriptions might be the reason causing such controversy [71].

The effect of corticosteroid use remains controversial. It was reported that corticosteroid use may increase the risk of fracture for patients with COPD [13–15]. However, a recent study reported that COPD was an independent risk factor for osteoporosis and fracture regardless of ICS use [72]. Moreover, cumulative ICS was reported not to increase the risk of fracture hospitalization [73]. Surprisingly, one study even showed that female COPD patients with ICS therapy exhibited dose-response protective effect on osteoporosis [69]. Similar controversy was also found in asthma. Monadi et al. [68] noted that the BMD in both the spinal cord and hip of asthma patients less than 50 years old under 6.5 years of treatment decreased significantly compared to the controls. On the other hand, Loke et al. [70] found that long-term use (≥12 months) of ICS in patients with asthma was not significantly associated with fracture and bone mineral density. Such controversies might be caused by differences in study design, duration of ICS use and cumulative dose, and frequency of systemic OCS prescriptions [71]. Frequent OCS administration may significantly increase the risk of osteoporosis and fracture. Investigations of ICS administration and osteoporosis or fracture for patients with noncystic fibrosis bronchiectasis are scant. It was reported that osteoporosis and osteopenia were prevalent in patients with bronchiectasis [74–76], which might be attributed to the intakes of proton pump inhibitor or inhaled corticosteroids although it was also associated with increased age and female gender [76].

#### 3.3.2. Prevalence of Fracture in Patients with Chronic Respiratory Disease Receiving OCS and ICS Therapy

Table 4 compares the prevalence of asthma (60.6% versus 68.2%, *p* < 0.001), COPD (64.7% versus 51.6%, *p* < 0.001), and bronchiectasis (5.8% versus 4.7%, *p* = 0.079) for patients with and without fracture receiving OCS and ICS. It was noted that the prevalence of asthma in patients with fracture was significantly lower than those without fracture, while COPD was more prevalent in patients with fracture than those without fracture. With regard to bronchiectasis, although it was more prevalent in patients with fracture, but not reaching significance, the inconsistency might be caused by a difficulty in differentiating skeletal effects of corticosteroid medications and COPD itself [67] and symptoms of COPD overlapped with asthma [77, 78] or bronchiectasis [79, 80] for some patients. The mortality rate of bronchiectasis was reported to be as high as 20.4% and increased to 55% if comorbid with COPD [81].

#### 3.3.3. Comorbidity of Patients with Chronic Respiratory Disease Receiving OCS and ICS Therapy

Diabetes (DM), end-stage renal disease (ESRD), and osteoporosis were shown to be comorbidities for patients with fracture who had been receiving OCS and ICS treatments. As presented in Table 4, the prevalence of DM (14.9% versus 12.4%, *p* < 0.05), ESRD (2.8% versus 1.4%, *p* < 0.001), and osteoporosis (7.1% versus 3.6%, *p* < 0.001) for patients with fracture was significantly higher than those without fracture. Bountiful literatures have shown the associations between diabetes and bone fracture. Forsen et al. [82] reported that women aged 50–74 years with type 2 diabetes for more than 5 years exhibited higher risk of hip fracture. It was also shown that patients with diabetes presenting diabetic retinopathy, advanced cortical cataract, longer acquired duration, and insulin treatment had higher risk of fracture [83]. Patients with ESRD were noted to have 4.4-fold risk of hip fracture than the general population [84]. Stehman-Breen et al. [85] reported that age, female, BMI, and peripheral vascular disease were associated with independent factors associated with hip fracture for patients with ESRD. The reason might be that renal osteodystrophy, amyloid, aluminum intoxication, and acidosis commonly found in ESRD patients receiving dialysis increased the risk of bone loss, resulting in hip fracture [84].

#### 3.3.4. Effectiveness of Clinical Decision Support Systems

Recently, we have conducted a prospective study to verify the effectiveness of a designed CDSS in ventilator weaning [19] and designed a predictive model for predicting erectile dysfunction using the Taiwan National Health Insurance Database [86]. For predicting successful ventilator weaning, a predictive sensitivity of 87.7% has been achieved by using CDSS, which is significantly higher (*p* < 0.01) than the weaning determined by physicians (sensitivity: 61.4%). Furthermore, the days using mechanical ventilator for the study group (38.41 ± 3.35) are significantly (*p* < 0.001) shorter than the control group (43.69 ± 14.89), with a decrease of 5.2 days in average, resulting in a saving of healthcare cost of NT$45,000 (US$1500) per patient in the current Taiwanese National Health Insurance setting [19]. The CDSS was demonstrated to be effective in identifying the earliest time of ventilator weaning for patients to resume and sustain spontaneous breathing, thereby avoiding unnecessary prolonged ventilator use and decreasing healthcare cost.

The CDSS for predicting ventilator weaning was designed based on the datasets collected in respiratory care centers which were more balanced; in contrast, the dataset adopted in this study was retrieved from NHIRD and was highly imbalanced. As shown in Table 2, although the CDSSs designed with our proposed method outperform the other methods [40], the performance is only fair with the accuracy, sensitivity, and specificity all lower than 70% and AUC smaller or a little greater than 0.7. As indicated in Table 7, CDSSs designed with integrated GA and SVM using different undersampling methods for obtaining balanced training sets and applying objective functions for tuning SVM parameters and selecting salient feature exhibit similar and satisfactory predictive performance with AUCs approximate to 0.75.

Although reaching statistically significant difference (*p* < 0.05) between fracture and nonfracture groups (Table 4), the variables regarding respiratory diseases (asthma and COPD) and other comorbidities (DM, ESRD, and osteoporosis) were not selected for designing the CDSSs. Moreover, as shown in Table 8, OCS and ICS variables, such as ICS_followup_days and ICS_exposed_days, which are statistically dependent with each other were selected. As argued in [53], filter methods like statistical analysis, *F* score, and entropy were not good at selecting salient features for CDSS design. Additionally, variables which are highly correlated can be used together to enforce the predictive performance of the CDSS [53].

Patients who had been prescribed with ICS might also be prescribed with oral corticosteroid (OCS) because of intermittent exacerbations. OCS administrated for treating acute exacerbation may have stronger effect than ICS on osteoporosis and fracture. Moreover, previous studies mainly focused on the effects of ICS on osteoporosis or fracture for patients with asthma and COPD, respectively. The effect of ICS on osteoporosis or fracture for patients with asthma-COPD and bronchiectasis-COPD overlap syndromes should also be considered. Future works will account for the effect of accumulated OCS and ICS dose, not just OCS or ICS dose, on osteoporosis or fracture. Patients with asthma-COPD overlap syndrome and bronchiectasis-COPD overlap syndromes will be separately considered for investigating the effect of OCS and ICS on fracture.

## 4. Conclusions

Based on the analytical results, it was concluded that long-term use of inhaled corticosteroids may induce osteoporosis and exhibit higher incidence of hip or vertebrate fractures. The designed CDSSs exhibited satisfactory performance in the prediction of fractures for patients who were prescribed with corticosteroids. We suggested that the accumulated dose of ICS and OCS therapies should be continuously monitored to prevent from exceeding the maximum dosage, especially for patients with older age and women after menopause.

## Figures and Tables

**Table 1:** Predictive performance by randomly undersampling the imbalanced training dataset.

Training subset	AC (%)	SE (%)	SP (%)	G-mean	AUC
1	66.32	63.02	66.53	0.6484	0.704
2	65.67	63.86	65.78	0.6481	0.698
3	67.75	60.92	68.18	0.6445	0.704
4	65.07	64.28	65.12	0.6470	0.700
5	65.42	62.18	65.62	0.6388	0.705
6	65.78	62.18	66.10	0.6411	0.713
7	67.75	60.92	68.18	0.6445	0.710
8	65.57	62.18	65.78	0.6396	0.710
9	67.29	65.54	67.29	0.6641	0.716
10	65.12	63.44	65.23	0.6433	0.709
Average	66.17	62.85	66.38	0.6439	0.707

**Table 2:** Comparisons of predictive performance between models designed based on the methods proposed in this study and other studies [40] with the CoIL challenge dataset [54].

CDSS model	AC (%)	SE (%)	SP (%)	G-mean	AUC
SVM + 100% oversampling	50.08	66.39	49.04	0.5706	0.5772
MLP + SMOTE	82.48	34.87	85.49	0.5460	0.6018
Hybrid SVM-MLP + 100% oversampling	52.1	63.87	51.36	0.5727	0.5762
LR + SMOTE	72.4	56.3	73.42	0.6429	0.6486
Hybrid SVM-LR + 100% oversampling	50.18	66.39	49.15	0.5712	0.5777
Decision tree (J48) + cluster-based kNN undersampling	55.68	68.50	54.86	0.6130	0.6020
One-sided selection + OB1	94.05	0	100	0	0.4997
One-sided selection + OB2	94.05	0	100	0	0.5000
GA-SVM + Rand undersampling with OB1	63.22	66.80	62.99	0.6487	0.7071
GA-SVM + Rand undersampling with OB2	62.92	67.64	62.62	0.6508	0.6885
GA-SVM + cluster-based kNN undersampling with OB1	65.72	65.12	65.76	0.6544	0.6997
GA-SVM + cluster-based kNN undersampling with OB2	62.67	65.96	62.46	0.6419	0.6599

**Table 3:** Comparisons of predictive performance between models designed based on the methods proposed in this study and the one-sided selection methods [43] with the WDBC dataset [54].

Group	Accuracy	Sensitivity	Specificity	G-Mean	AUC
GA-SVM + Rand undersampling with OB1	97.64	97.64	97.64	0.9764	0.9915
GA-SVM + Rand undersampling with OB2	97.40	97.64	97.16	0.9740	0.9970
GA-SVM + cluster-based kNN undersampling with OB1	98.11	98.11	98.11	0.9811	0.9945
GA-SVM + cluster-based kNN undersampling with OB2	97.40	96.22	98.58	0.9739	0.9965
One-sided selection with OB1	97.84	97.97	97.64	0.9780	0.9956
One-sided selection with OB2	97.49	96.22	98.26	0.9723	0.9984

**Table 4:** Comparisons of demographic characteristics, comorbid respiratory diseases, and other comorbidities between patients with and without fracture.

	Fracture		*p* value
	No (*N* = 10511)	Yes (*N* = 1134)	
*Sex*			0.001
Men	6211 (59.1%)	614 (54.1%)	
Women	4300 (40.9%)	520 (45.9%)	
*Age (mean ± SD, year)*	58.5 ± 18.1	70.5 ± 12.5	<0.001
*Age (year)*			
20–40	1985 (18.9%)	41 (3.6%)	
41–50	1364 (13.0%)	35 (3.1%)	
51–64	2456 (23.4%)	182 (16.0%)	
≥65	4706 (44.8%)	876 (77.2%)	
*Chronic respiratory diseases*			
Asthma	7172 (68.2%)	687 (60.6%)	<0.001
COPD	5424 (51.6%)	734 (64.7%)	<0.001
Bronchiectasis	489 (4.7%)	66 (5.8%)	0.079
*Other comorbidities*			
DM	1302 (12.4%)	169 (14.9%)	0.015
Cancer	464 (4.4%)	53 (4.7%)	0.687
Liver cirrhosis	88 (0.8%)	13 (1.1%)	0.286
ESRD	151 (1.4%)	32 (2.8%)	<0.001
Osteoporosis	377 (3.6%)	81 (7.1%)	<0.001

**Table 5:** Association between prescribed oral or intravenous corticosteroid and fracture.

	Fracture		*p* value
	No (*N* = 10511)	Yes (*N* = 1134)	
*Medication (oral or intravenous corticosteroids)*	10511 (90.3%)	1134 (9.7%)	
*Cumulative dose (mg)*			<0.001
Mean ± SD	1345.77 ± 2831.30	2355.86 ± 4049.43	
Median	390	810	
*Mean follow-up daily dose (mg/d) (cumulative dose/follow-up duration)*			0.979
Mean ± SD	59.74 ± 218.39	59.56 ± 285.58	
Median	4.54	3.275	
*Mean exposed daily dose (mg/d)*			0.001
*(Cumulative dose/cumulative exposed duration)*			
Mean ± SD	67.65 ± 235.62	94.04 ± 314.98	
Median	13.45	15	
*Duration (day), median (range)*			
Follow-up duration	706.77 ± 907.35	999.65 ± 997.09	<0.001
Median	240	731.0	
Exposed duration	90.11 ± 194.80	154.04 ± 320.91	<0.001
Median	28	38.5	

**Table 6:** Association between prescribed inhaled corticosteroid and fracture.

	Fracture		*p* value
	No (*N* = 10511)	Yes (*N* = 1134)	
*Medication (inhaled corticosteroids)*	10511 (90.3%)	1134 (9.7%)	
*Cumulative dose (mg)*			<0.001
Mean ± SD	171.97 ± 318.13	230.14 ± 442.76	
Median	45	54	
*Mean follow-up daily dose (mg/d) (cumulative dose/follow-up duration)*			<0.001
Mean ± SD	1.00 ± 3.91	0.44 ± 2.31	
Median	0.07	0.05	
*Mean exposed daily dose (mg/d)*			0.261
*(Cumulative dose/cumulative exposed duration)*			
Mean ± SD	0.55 ± 1.35	0.51 ± 0.81	
Median	0.42	0.39	
*Duration (day), median (range)*			
Follow-up duration	1370.91 ± 1097.76	1777.17 ± 1039.82	<0.001
Median	1239.0	1862.0	
Exposed duration	342.35 ± 503.32	453.79 ± 584.65	<0.001
Median	121.0	199.0	

**Table 7:** Comparisons of predictive performance among different sampling methods.

Group	Training phase			Testing phase				
	AC (%)	SE (%)	SP (%)	AC (%)	SE (%)	SP (%)	G-mean	AUC
Random undersampling with OB1	68.57	74.82	62.32	63.16	77.00	62.36	0.6929	0.7590
Random undersampling with OB2	68.92	68.92	68.92	68.30	70.03	68.20	0.6909	0.7495
Clustering-based kNN undersampling with OB1	71.25	75.66	66.84	63.40	76.19	62.20	0.6884	0.7526
Clustering-based kNN undersampling with OB2	71.25	71.25	71.25	67.28	69.84	67.00	0.6840	0.7515
One-sided selection + OB1	77.80	82.59	35.62	94.54	0	100	0	0.7007
One-sided selection + OB2	89.75	1.41	99.79	71.43	41.98	73.13	0.5541	0.6626

**Table 8:** Optimal SVM parameters and selected features for CDSS design.

Undersampling + objective function	Random + OB1	Random + OB2	Clustered + OB1	Clustered + OB2	One sided + OB1	One sided + OB2
*α*	3.3	11.9	22.0	3.6	25.0	16.8
*γ*	−5.6	−6.8	−22.0	−2.8	−25.0	−13.5
Sex	x	x	x	x	x	x
Age	x	x	x	x		x
Asthma						
COPD						
Bronchiectasis						
DM						
Cancer						
Liver cirrhosis						
ESRD						
Osteoporosis						
OCS_followup_days	x		x		x	x
OCS_exposed_days	x	x	x	x		x
ICS_followup_days	x	x	x	x		x
ICS_exposed_days	x	x	x	x		x
OCS_dose	x	x	x			
ICS_dose	x		x	x		
OCS_follow_daily_dose	x			x		x
OCS_exposed_daily_dose		x	x			
ICS_follow_daily_dose	x	x	x	x		x
ICS_exposed_daily_dose						
